# Relative rates of cancers and deaths in Australian communities with PFAS exposure.

**DOI:** 10.23889/ijpds.v7i3.1977

**Published:** 2022-08-25

**Authors:** Hsei Di Law, Bruce Armstrong, Catherine D'Este, Rose Hosking, Kayla Smurthwaite, Susan Trevenar, Nina Lazarevic, Martyn Kirk, Rosemary Korda

**Affiliations:** 1 Australian National University; 2 The University of Western Australia; 3 Sax Institute

## Objectives

The use of firefighting foam containing per- and polyfluoroalkyl substances (PFAS) has resulted in environmental contamination in three Australian communities. We examined whether people who had lived in these communities had higher rates of selected cancers and causes of deaths than those who had lived in comparison areas without known contamination.

## Approach

The three exposure areas of interest were in Katherine (NT), Oakey (Qld) and Williamtown (NSW). We identified those who ever lived in exposure areas by linking street addresses in these areas to addresses collected in Medicare (1983-2019)—a consumer directory for Australia’s universal healthcare system. We also identified a sample of those who had lived in selected comparison areas. Exposed and comparison populations were then linked to Australia’s national cancer and death registries. We estimated standardised incidence ratios (SIRs) for 23 cancers, four causes of death and three control outcomes, adjusting for sex, age and calendar time of diagnosis.

## Results

We observed higher rates of prostate cancer (SIR = 1·76, 95% confidence interval (CI) 1·36–2·24) in Katherine; laryngeal cancer (SIR = 2·71, 95% CI 1·30–4·98), kidney cancer (SIR = 1·82, 95% CI 1·04–2·96) and coronary heart disease (CHD) mortality (SIR = 1·81, 95% CI 1·46–2·33) in Oakey; and lung cancer (SIR = 1·83, 95% CI 1·39–2·38) and CHD mortality (SIR = 1·22, 95% CI 1·01–1·47) in Williamtown. We also saw elevated SIRs for control outcomes—outcomes not known or thought to be associated with PFAS exposure. SIRs for all other outcomes and overall cancer were similar across exposure and comparison areas.

## Conclusion

There was limited evidence to support an association between PFAS exposure and risk of cancer. There was modest evidence of an association with CHD mortality, which merits further study given the links between PFAS and elevated blood lipids.

